# Advancing Reduction Mammaplasty Surgery: Advancements and Outcomes with Tumescent Local Anaesthesia

**DOI:** 10.1007/s00266-024-04412-4

**Published:** 2024-09-29

**Authors:** Giovanni Arrica, Matilde Tettamanzi, Federico Ziani, Edoardo Filigheddu, Claudia Trignano, Corrado Rubino, Emilio Trignano

**Affiliations:** 1 Plastic Surgery Unit, Department of Surgical, Microsurgical and Medical Sciences, University of Sassari, Sassari, Italy; 2 Department of Biomedical Sciences, University of Sassari, Sassari, Italy

**Keywords:** Reductive mammoplasty, Breast, Tumescent local anaesthesia, Macromastia

## Abstract

**Background:**

Tumescent local anaesthesia (TLA) is a method of anaesthesia used for surgical procedures that involves the infusion of a saline solution containing lidocaine, sodium bicarbonate, and epinephrine. This anaesthetic technique is designed to achieve both vasoconstriction and anaesthesia. In this article, we present a modified TLA protocol specifically adapted for reduction mammaplasty, based on an analysis of clinical case histories collected over the past few years.

**Methods:**

During the period from 2012 to 2022, we performed a reduction mammaplasty procedure in 120 patients employing tumescent local anaesthesia (TLA). The composition of the tumescent solution included 25 mL of 2% lidocaine, 8 mEq of sodium bicarbonate, and 1 mL of epinephrine (1 mg/1 mL) in 1000 mL of 0.9% saline solution. The solution was injected diffusely throughout the mammary gland.

**Results:**

The average volume of tumescent solution infiltrated during TLA was 350 mL per breast. There were no cases of adrenaline or lidocaine toxicity, and no patients required conversion to general anaesthesia. No patient received sedation. Patients reported no pain or discomfort during pre-operative infiltration or during surgery.

No reinterventions were necessary because of short-term complications. Among the complications, there were 4 cases of hematoma (3,3%), 3 cases of seroma (2,55%), 10 cases of wound dehiscence (8,3%), 5 cases of asymmetry (4,1%), 9 cases of T-junction breakdown (7,5%), 2 cases of (partial) nipple necrosis (1,6%), and 3 cases of liponecrosis (2,5%).

No cases of infection or total nipple-areola loss were reported. The follow-up period was between 30 days and 1 year.

**Conclusions:**

Reduction mammaplasty is a viable surgical option for women with macromastia seeking to enhance their physiognomy. It is imperative that patients fully understand the potential benefits and risks associated with the procedure and consult with healthcare professionals specialising in this field.

The use of tumescent local anaesthesia (TLA) has been confirmed as a safe and effective methodology to perform reduction mammaplasty, ensuring adequate pain control with minimal post-operative complications and resulting in a high degree of patient satisfaction.

**Level of Evidence IV:**

This journal requires that authors assign a level of evidence to each article. For a full description of these Evidence-Based Medicine ratings, please refer to the Table of Contents or the online Instructions to Authors www.springer.com/00266.

## Introduction

Reduction mammaplasty, a surgical procedure aimed at reducing the size and reshaping the breasts, is commonly performed to alleviate physical discomfort, functional impairment, and psychological distress associated with macromastia. While traditional reduction mammaplasty techniques have demonstrated efficacy in achieving desirable aesthetic outcomes and improving patients’ quality of life, advancements in anaesthesia protocols have revolutionised the perioperative experience and surgical outcomes [1]. Tumescent local anaesthesia has emerged as a promising alternative to general anaesthesia in reduction mammaplasty surgery, offering several potential advantages including improved safety, enhanced post-operative pain management, and reduced systemic complications related to general anaesthesia [2]. This technique involves the infiltration of a large volume of diluted local anaesthetic solution, typically containing lidocaine and epinephrine, into the breast tissue and surrounding areas [3]. The tumescent solution not only provides effective anaesthesia but also serves as a vasoconstrictor, minimising intraoperative bleeding and facilitating surgical dissection [4]. Despite the growing interest in tumescent local anaesthesia for reduction mammaplasty, comprehensive scientific investigations evaluating its efficacy, safety profile, and perioperative outcomes are relatively scarce. Thus, this study aims to address this gap by systematically examining the clinical outcomes and patient-reported experiences associated with reduction mammaplasty performed under tumescent local anaesthesia. By elucidating the benefits and potential limitations of tumescent local anaesthesia in reduction mammaplasty surgery, this research endeavours to contribute to the optimisation of surgical techniques and perioperative care protocols. Through rigorous evaluation of surgical outcomes, complication rates, and patient satisfaction measures, this study aims to provide valuable insights that can inform clinical decision-making and enhance the overall management of macromastia.

## Materials and Methods

Between 2012 and 2022, a total of 120 individuals underwent corrective surgery for reduction. All operations took place at a certified outpatient facility. The surgical team comprised a plastic surgeon with board certification, an assistant surgeon, an operating room nurse, and a certified anaesthesiologist. The mean age of the patients was 47 years, with a range spanning from 18 to 75 years, while the mean body weight and body mass index (BMI) averaged at 65 kg and 27,3 kg/m^2^, respectively. Patients received comprehensive information about the procedure, its indications, and potential complications, such as post-operative bleeding, scar retraction, asymmetry, wound dehiscence, liponecrosis, and seroma formation. Prior to surgery, patients underwent standard pre-operative tests, including blood work, cardiac evaluation, and breast ultrasound. All patients met the American Society of Anesthesiologists (ASA) criteria for either status I or II [5]. Exclusion criteria included ASA status III or higher, as well as BMI considerations (BMI > 35). The patients’ characteristics are outlined in Table 1. Pre-operative drawings were made with the patient in the standing position and completed in the prone position.

**Table 1:** Patients and surgery’s characteristics

Characteristic	Values
Mean age	47 years (range 18–75 years)
Mean BMI	27,3 kg/m^2^ (range 24–29 kg/m^2^)
Mean body weight	65 kg (range 59–76 kg)
Average volume of TLA per breast	350 mL (range 250–500 mL)
Average time interval from solution infiltration to skin incision	20 min
Volume of mammary gland removed	From 160 to 250 grams
Mean duration of surgery	80 min (range 60–90 min)

The midsternal line, breast width, submammary fold, projection of the submammary fold to the midsteral line, and lateral thoracic borders are drawn. Then, after the standard drawings, the design and planning of the reduction mammaplasty was done [6]. All surgeries were performed with a superomedial dermoglandular pedicle technique and an inverted T-shaped scar. In relation to the expected residual breast volume following surgery, the mean length of the SF (sternal notch to inframammary fold) was 21 cm, the NF (nipple to inframammary fold) was 6 cm, and the areola diameter was 4 cm. Photographic documentation was acquired before the patient entered the operating room and was positioned supine on the operating table. Medications affecting platelet function were discontinued 5–7 days before surgery or substituted with suitable alternatives. A certified anaesthesiologist was present at all times during surgery. Each patient received a peripheral intravenous line. Cardiac function, blood pressure, and oxygen saturation were monitored at all times during surgery and recovery.

No patient received sedation. Breast anaesthesia involved the use of tumescent solution, consisting of 25 mL of 2% lidocaine, 8 mEq of sodium bicarbonate, and 1 mL of epinephrine (1 mg/1 mL) in 1000 mL of 0.9% saline solution, with a total volume of 350 mL per breast. The standard lidocaine dosage ranged from 1.4 to 2.8 mg/kg. The amount of tumescent solution administered varied based on chest size and BMI, ensuring breast turgidity and minimising the risk of drug toxicity, particularly in patients with smaller chests and glandular tissue and lower body weights. The volume of mammary gland removed was between 160 and 250 grams.

### Surgical Technique

Following pre-operative markings, the cutaneous incision site was infiltrated with 1% lidocaine containing 1:100,000 epinephrine. A spinal needle connected to a peristaltic infiltration pump was used to diffusely inject local tumescent anaesthetic into the mammary gland. The infusion was halted upon reaching an average volume of 350 mL per breast. Glandular infiltration ensured thorough anaesthesia through direct contact. The initial incision was made 20 min later to allow for the full effect of epinephrine and lidocaine. After this time, the superomedial dermoglandular pedicle is deepithelialised with a no. 10 blade. Incisions are first made with a no. 15 blade and carried on with monopolar cautery. The gland is then removed en bloc with the skin overlying the lateral and inferior dermoglandular tissue, reaching down to the underlying fascia. After the removal of the mammary gland, the superomedial pedicle is lifted from the underlying fascia and rotated 90° medially (clockwise on the right breast, repositioning the areola’s 9 o’clock point at 12 o’clock, the 3 o’clock point at 6 o’clock, and counterclockwise on the left breast, repositioning the areola’s 3 o’clock point at 12 o’clock, the 9 o’clock point at 6 o’clock, etc.). Fixing following rotation is performed with an intermittent suture (Vicryl 2-0). Closure was performed in planes: the glandular pillars were sutured with intermittent suture (Vicryl 2-0), then the inner subcutaneous layer with delayed intermittent absorbable suture (Monocryl 3-0) and eventually the skin with subcutaneous absorbable suture (Monocryl 4-0). Throughout the procedure, meticulous haemostasis was maintained. Drains were not utilised. After a 4-h observation period, patients were discharged. Depending on allergy status, an oral antibiotic regimen (Amoxicillin 875 mg/Clavulanic Acid 125 mg or Ciprofloxacin 500 mg twice daily) was prescribed for 5 days, with post-operative follow-ups scheduled at 1 day, 1 week, 2 weeks, 1–3–6 months, and 1 year.

## Results

Over the course of a 10-year interval, a comprehensive analysis was conducted on a cohort comprising 120 individuals who had undergone reductive mammaplasty procedures, exclusively employing the tumescent local anaesthesia technique. A mean volume of 350 mL of tumescent solution was infiltrated, with a range between 250 and 500 mL, and no instances of adverse effects related to adrenaline or lidocaine toxicity were documented. General anaesthesia conversion was never necessitated during any of the procedures. The average time interval from solution infiltration to the initiation of skin incision was determined to be at least 20 min, a parameter determined through collaborative assessment by the surgical team and anaesthesiologist. Departing from this time threshold resulted in heightened patient discomfort, while exceeding it conferred no discernible benefits to the patient. During the surgical intervention, no reports of pain emerged during the skin incision or the removal of the mammary gland. The mean duration of surgery employing the TLA technique ranged from 60 min to 90 min, excluding bilateral infiltration and waiting periods. Within the scope of post-operative complications, comprising there were 4 cases of hematoma (3,3%), 3 cases of seroma (2,55%), 10 cases of wound dehiscence (8,3%), 5 cases of asymmetry (4,1%) 9 cases of T-junction breakdown (7,5%), 2 cases of (partial) nipple necrosis (1,6%), and 3 cases of liponecrosis (2,5%). No cases of infection or total nipple-areola loss were reported (Table 2).

**Table 2:** Post-operative complication rate after breast reduction in 120 patients

Complications	Patients	%
Hematoma	4	3,3
Seroma	3	2,5
Wound dehiscence	10	8,3
Asymmetry	5	4,1
T-junction breakdown	9	7,5
Liponecrosis	3	2,5
Partial nipple necrosis	2	1,6
Need for reintervention	0	0

**Fig. 1:**
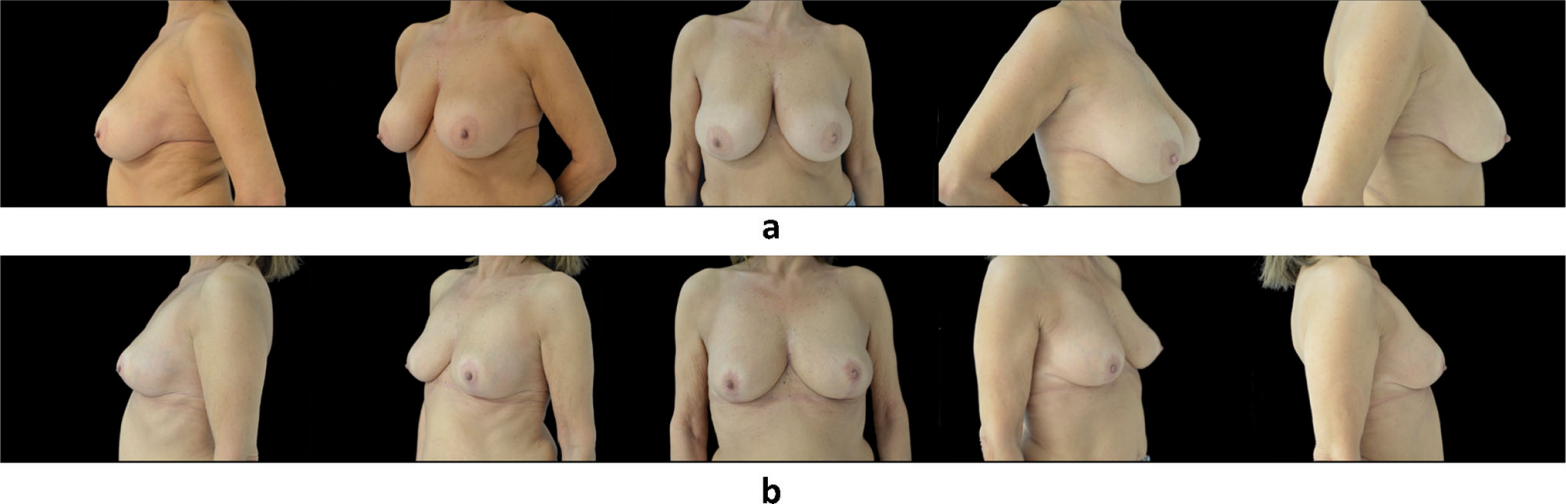
**a** Pre-operative view. **b** Post-operative view after 1 year

**Fig. 2:**
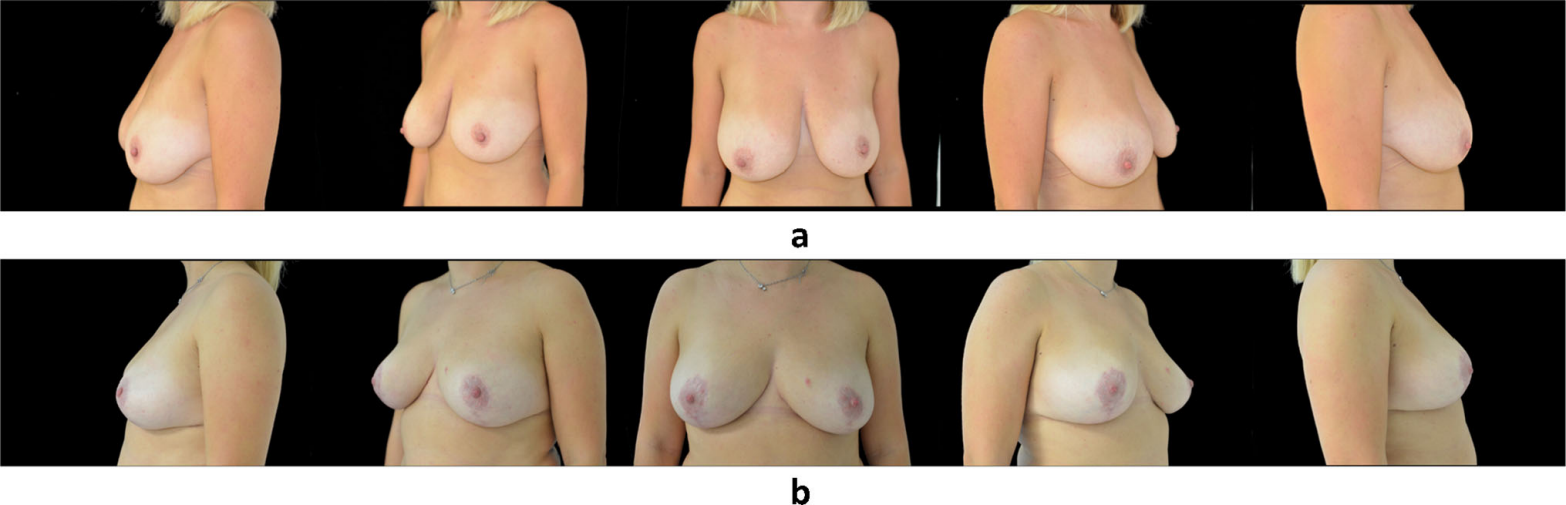
**a** Pre-operative view. **b** Post-operative view after 1 year

Patients expressed overall satisfaction with the TLA procedure, reporting no discomfort during the pre-operative infiltration or throughout the entire surgical process. Most patients conveyed contentment with the aesthetic outcomes at the 1-year follow-up. A satisfaction survey was administered 3 months post-surgery, enabling patients to rate their pain management and satisfaction with their aesthetic results on a scale ranging from “unsatisfactory” to “excellent”. The majority of patients expressed high levels of satisfaction. Importantly, all patients were subject to a follow-up period exceeding 1 year (Figs. 1, 2).

## Discussion

In this article, we present our 10-year experience with reduction mammaplasty, a procedure performed in 120 patients using the TLA technique.

The TLA technique (modified tumescent local anaesthesia) for reduction mammaplasty operations symbolises a new step for this type of surgery [2]. The use of local anaesthesia for breast surgery has been described in the literature and there are several procedures that can be performed using this anaesthetic technique [7]. The progression in studies concerning this anaesthetic technique, considering its potential for minimising complications and its less invasive nature compared to general anaesthesia, supports the hypothesis that it can be used as an excellent alternative in surgery [8, 9].

Tumescent anaesthesia describes the practice of injecting a diluted solution of local anaesthetic combined with epinephrine and sodium bicarbonate into the subcutaneous tissue until it becomes firm and taut (tumescent) [10]. They are usually injected deep into the subcutaneous tissue. In addition to the anaesthetic action, this technique offers the advantage of performing tissue dissection (hydrodissection); by combining it, blood loss is reduced through the vasoconstriction induced by adrenaline together with the hydrostatic effects of injecting large volumes that tamponade local blood vessels. Adrenaline also significantly prolongs the blockade resulting in excellent post-operative anaesthesia and analgesia. Sodium bicarbonate accelerates the onset of blockade and reduces the pain associated with the injection of local anaesthetic [11].

In current plastic surgery practice, it can be used in various combinations, alone or with varying levels of sedation, to provide intraoperative analgesia. Potential benefits include reduced narcotic use, less need for sedation, faster recovery, and earlier discharge, resulting in cost savings. In addition, a reduction in the rate of venous thromboembolism associated with general anaesthesia may be considered [12].

Multiple studies have shown that intravenous anaesthesia is associated with a lower incidence of post-operative nausea and vomiting. [13, 14]; however, it should be noted that propofol and remifentanil anaesthesia tends to be more expensive than alternative methods [15, 16].

Tahiri et al. [17] published a review comprising five studies that compared the incidence of post-operative nausea and vomiting (PONV) between thoracic paravertebral block (TPVB) and general anaesthesia (GA) for breast surgery. The incidence of PONV in patients treated with TPVB ranged from 0 to 23.5%, which was lower than the 6.7–40% observed in patients undergoing GA. Eldor et al. [18] conducted a comparison between breast augmentation performed under general anaesthesia (GA) and monitored anaesthesia care (MAC). Vomiting was more frequently observed in the GA group, with 84.1% of patients in the MAC group experiencing no vomiting at all, compared to only 60.9% in the GA group.

Jabs et al. [19] investigated the combination of general anaesthesia with intraoperative infiltration of a standard tumescent solution (consisting of 1 mL lactated Ringer’s, 50 mL 1% lidocaine and 1 mL 1:1000 epinephrine). The results showed a reduction in post-operative pain and a significant decrease in the use of analgesics in the immediate perioperative period. However, the use of general anaesthesia still results in a longer post-operative recovery time. This type of anaesthesia may allow reduction mammaplasty to be performed under intravenous (IV) sedation instead of general anaesthesia. In Zukowski et al. study [20], intravenous fentanyl and midazolam were used with TLA in 50 patients and breast tissue resections of 516–2948 g were performed with an average operating time of 3 h.

In our clinical practice, reduction mammaplasty required approximately 350 mL of solution per breast, an amount well below the established safety levels for TLA in adult patients (55 mg/kg) [21, 22].

Lidocaine toxicity is closely related to its plasma levels, which may vary due to rapid systemic absorption, impaired hepatic metabolism, or drug interactions. It is therefore essential to monitor the patient during infiltration. Moreover, it is also important to start the injection of the tumescent solution slowly while palpating the breast to ensure that the solution is distributed in the correct tissue plane. The use of epinephrine induces vasoconstriction, thereby reducing blood loss and bleeding during surgery [4]. However, it is essential to perform careful haemostasis as soon as the effect of epinephrine wears off to prevent post-operative bleeding. If adequate haemostasis is achieved, the use of drains will not be necessary, as is the case in our practice [23].

This type of anaesthesia can be used in patients who refuse general anaesthesia. It is also indicated in patients with myasthenia gravis; this approach can be used to avoid general anaesthesia and minimise the use of sedative or potentially aggravating medications. Amide local anaesthetics are recommended, as the metabolism of ester anaesthetics may be impaired by the patient’s regular anticholinesterase medications [24].

Recovery from tumescent local anaesthesia (TLA) is generally quicker than from general anaesthesia because the latter requires the administration of strong anaesthetics that induce loss of consciousness and deep muscle relaxation. This takes time for the effects of the drugs to wear off, which can prolong recovery. TLA, on the other hand, only anaesthetises the specific area affected without affecting the patient’s level of consciousness. In addition, the drugs used in TLA (such as lidocaine and adrenaline) have minimal systemic effects compared to those used in general anaesthesia. Another advantage of TLA is that it does not affect respiratory function, reducing the risk of complications such as hypoventilation or aspiration, which can delay post-operative recovery [25]. General anaesthesia often requires intubation and mechanical ventilation, with the associated risk of post-operative respiratory complications. Surgery performed with the TLA technique allows early mobilisation, which, together with the lack of muscle relaxation associated with general anaesthesia, reduces post-operative complications such as deep vein thrombosis [26]. No muscle relaxants were required during the procedure, maintaining muscle tone was not an obstacle [27]. As the volume of the injected solution significantly alters the shape of the breasts, careful pre-operative marking is essential to ensure an accurate aesthetic result. In addition, patients should be thoroughly informed that post-operative breast swelling is to be expected and will persist for several weeks, initially affecting the final aesthetic result. This preparatory step is crucial for achieving optimal surgical results and for giving the patient realistic expectations regarding the immediate post-operative appearance. A comprehensive evaluation of results requires a minimum of 3 months post-operatively. This period allows for the full resolution of tissue imbibition, a process triggered by the significant volume of solution injected. During this period, the gradual resolution of tissue swelling and the stabilisation of breast contours occur, allowing a more accurate assessment of the final aesthetic results.

The complications observed in our cohort are consistent with those reported in the literature, demonstrating similar incidence rates and clinical manifestations. Among the complications observed, T-junction breakdown was predominant, not due to excessive dermoglandular tissue removal, but rather to tissue imbibition resulting from the instillation of tumescent local anaesthesia (TLA); however, the extent of this complication was minimal. This phenomenon highlights the importance of understanding the complex interplay between surgical technique and anaesthetic administration in reducing post-operative complications. The cases of post-operative asymmetry were minor and did not require reintervention to correct, and were mainly in patients who already had some degree of breast asymmetry prior to surgery.

Among the complications observed there were seroma and haematoma, which also occur in other types of breast surgery [28–30]. Both complications were minor and resolved without the need for reoperation. Another minor complication observed in our study was the occurrence of dystrophic scarring associated with delayed wound closure. In response to these cases, we introduced the use of a polyurethane dressing as an adjunctive measure to promote and accelerate the wound healing process [31]. None of the complications encountered in our patient cohort required reintervention; when complications did occur, they were efficiently managed by medical treatment.

## Conclusion

Reduction mammaplasty is a useful procedure for patients who wish to have smaller breasts or who have breast volume problems. Before the procedure, it is important for the patient to discuss with the plastic surgeon all the potential benefits and risks of the procedure. Tumescent local anaesthesia is a safe and effective method of performing breast reduction surgery. Patients have expressed satisfaction with the technique and there have been no documented intraoperative complications. However, we continue to recommend the presence of a board-certified anaesthetist for proper patient selection and contingency planning in the event of significant anaesthesia-related complications.
